# Influence of a novel suturing technique on periodontal health of mandibular second molar following impacted third molar surgery: a split-mouth randomized clinical trial

**DOI:** 10.1186/s40902-022-00342-w

**Published:** 2022-03-22

**Authors:** Mohammad Esmaeelinejad, Mohammadhossein Mansourian, Farzad Aghdashi

**Affiliations:** 1 Department of Oral and Maxillofacial Surgery, School of Dentistry, Semnan University of Medical Sciences, Semnan, Iran; 2 Iranian Association of Oral and Maxillofacial Surgeons, Tehran, Iran; 3 Private Practice, Tehran, Iran; 4 Department of Oral and Maxillofacial Surgery, School of Dentistry, Shahid Beheshti University of Medical Sciences, Tehran, Iran

**Keywords:** Oral surgery, Periodontal pocket, Sutures, Third molar

## Abstract

**Background:**

Surgical extraction of the third molar is the most common surgical procedure in the oral surgery field and is associated with several complications. This study aimed to compare the effects of a newly presented suturing technique with the routine suture after surgical removal of the third molar on the postoperative complications.

**Materials and methods:**

This randomized clinical trial was designed as a split-mouth double-blinded investigation. Twenty patients were involved in the current study. After the surgical removal of the third molar, the new suturing technique was used to close the wound on one side of the patient randomly (case side), and the other side was sutured by the routine simple interrupted stitches (control side). Pain, edema, trismus, pocket depth, and the attachment loss of the distal of the second molar were assessed following the surgery. The data were statistically analyzed and compared between the sides.

**Results:**

Pain and edema following the surgery in the control side were significantly less than in the case side. The pocket depth and the gingival attachment loss of the distal aspect of the second molar in the case side were significantly less than in the control side. No case of dry socket was observed in the case side.

**Conclusion:**

It seems that the newly presented suturing technique is able to keep the wound margins close to each other and may be helpful in reducing the periodontal complication of the second molar following the surgical removal of the impacted third molars.

## Background

The third molar is the most frequently impacted tooth, and surgical removal of this tooth is the most common oral surgery [1]. The etiologies of third molar impaction are various, and the frequency of impaction is different regarding the genetic and environmental factors [2]. Surgical extraction of impacted third molar surgery is indicated due to several complications such as periodontal problems of the second molar, development of pathologic lesions, and possibility of mandibular angle fracture [3]. Therefore, management of impacted third molars is an important and challenging issue among dentists and maxillofacial surgeons.

On the other hand, surgical extraction of impacted third molar may be associated with some complications like injury to the trigeminal nerve, trismus, and periodontal problems of the second molar [4, 5]. These complications could affect the patient’s quality of life, and preventing these complications is a challenge in the oral surgery field. Some of these complications are reduced by time; however, periodontal problems of the second molar that occur following the third molar surgery are a long-standing defect. Despite pain and hemorrhage following impacted third molar surgery, periodontal defects of the second molar are not obvious to the patient or ignored as a serious problem. Gingival pocket formation and attachment loss on the distal of the second molar provide an environment for bacterial plaque and result in losing the second molar in the future [6, 7].

Several investigations have introduced specific flap designs to reduce the risk of periodontal defects following third molar surgery [8, 9]. Some other researches have revealed special suturing techniques are capable to prevent attachment loss after impacted third molar extraction [2, 10]. Anchor suturing technique [2] and modified triangular flap [11] are the two indicated methods in the literature that may be able to prevent gingival pocket formation and attachment loss after third molar surgery. However, conflicting results extracted from these investigations have allowed the periodontal problem of the second molar tooth to remain a dilemma following third molar surgery [2, 10].

This study aimed to propose a newly presented suturing technique and assess its effectiveness in reducing postoperative complications of impacted third molar extraction.

## Materials and methods

This randomized double-blind clinical trial was executed in 2019 in the dental school of Semnan University of Medical Sciences, Iran. Twenty patients were involved in the current study.

### Surgical procedure

The random allocation was performed by block randomization to divide both sides of twenty patients into two groups (40 cases). In the first group, the first surgery was performed on the right side of the mandible, and in the second group, the first surgery was performed on the left side of the mandible. All surgeries were performed by an experienced maxillofacial surgeon. The impact pattern of the third molars was the same in each case (right side and the left side). All patients were candidates for mandibular third molar surgical removal; these teeth were categorized in group C according to Pell and Gregory’s classification [12] and had an extraction difficulty of 7–10 grade according to Pederson’s scale [13]. The second molars were periodontally intact, and neither periodontal pocket nor attachment loss was observed before the surgery. All the patients were healthy according to their medical history, and no systemic diseases were reported. All of them were non-smokers, and the female patients did not use any oral contraceptives. The type of stitches in the first surgery was randomly selected and was not shown to the surgeon before starting the stitches and completing the surgical procedure. The patient and the evaluator were blind to the stitches, and the study design was double-blind.

The patient used a 0.2% chlorhexidine mouthwash for 1 min before starting the surgery. Conventional alveolar inferior anesthesia (2 ml) and long buccal (1 ml) were performed with 2% lidocaine and 1:100,000 epinephrine. The full-thickness mucoperiosteal flap designed by a surgical blade (Moris, Humburg, Germany) started from the buccal region of the first molar and passed through the gingival sulcus of the teeth to the line angle of the second molar and from there to the lateral, up, and back and extends on the anterior ramus. The flap was elevated by a periosteal elevator (Juya Instruments PVT, Tehran, Iran), and an osteotomy was performed by a surgical handpiece (NSK, Tokyo, Japan). If needed, the tooth crown was sectioned by a drill (Hager & Meisinger GmbH, Neuss, Germany), and all parts of the tooth were removed.

**Fig. 1:**
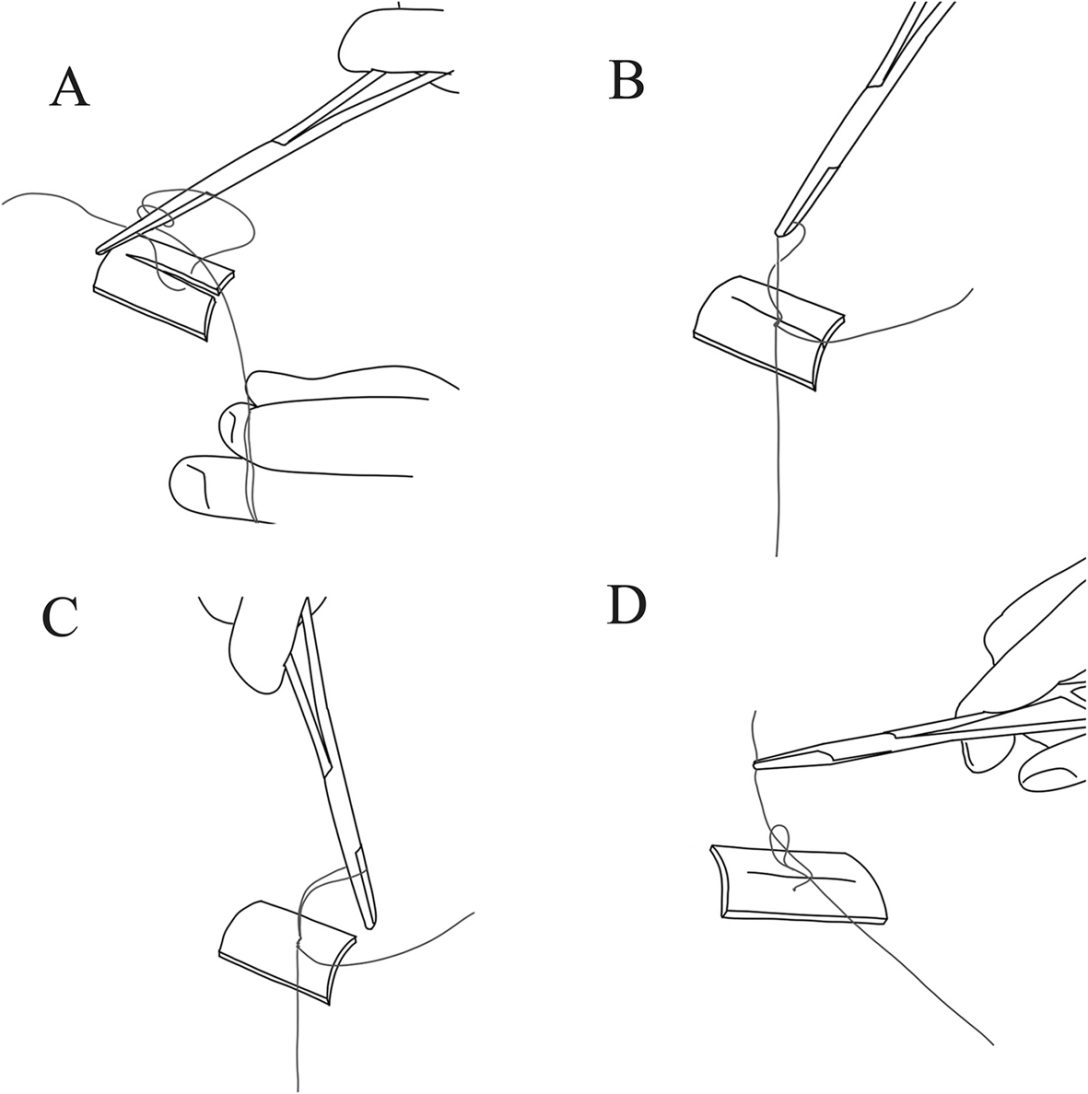
Schematic view of the new suturing technique. **A** The free end of the suture should be wrapped around the needle holder twice. **B** The middle of the short end of the suture is grasped by the needle holder. **C** The short end of the suture is passed through the loop not completely to make a bow tie. **D** The needle holder is passed through the bow tie to grasp the free end of the suture and pull it over to complete the knot. This procedure is repeated one more time

The distal wedge was removed from the gingival of the second molar to prevent future pocket occurrence. The follicle of the third molar was removed, and the surgical site was irrigated by 10 ml of 0.9% saline. The flap was returned to its site and sutured by 3/0 vicryle stiches (SUPA Inc., Tehran, Iran). In the control side of the patient, two simple interrupted stitches were used to close the wound. On the other side, the new suturing technique was applied to suture the surgical side. The schematic view of this newly presented suturing technique is shown in Fig. 1. The interval between the surgeries was 4 weeks for each individual. The patient and the assessor were blinded to the suturing technique. No hemostatic substance was used at the surgical site. Chlorhexidine 0.12% twice daily, amoxicillin 500 mg 3 times daily for 5 days (in case of sensitivity, clindamycin 300 mg every 8 h for 5 days), and acetaminophen/codeine 325/10 mg every 6 h for 48 h were prescribed for the patient.

### Evaluation

**Fig. 2:**
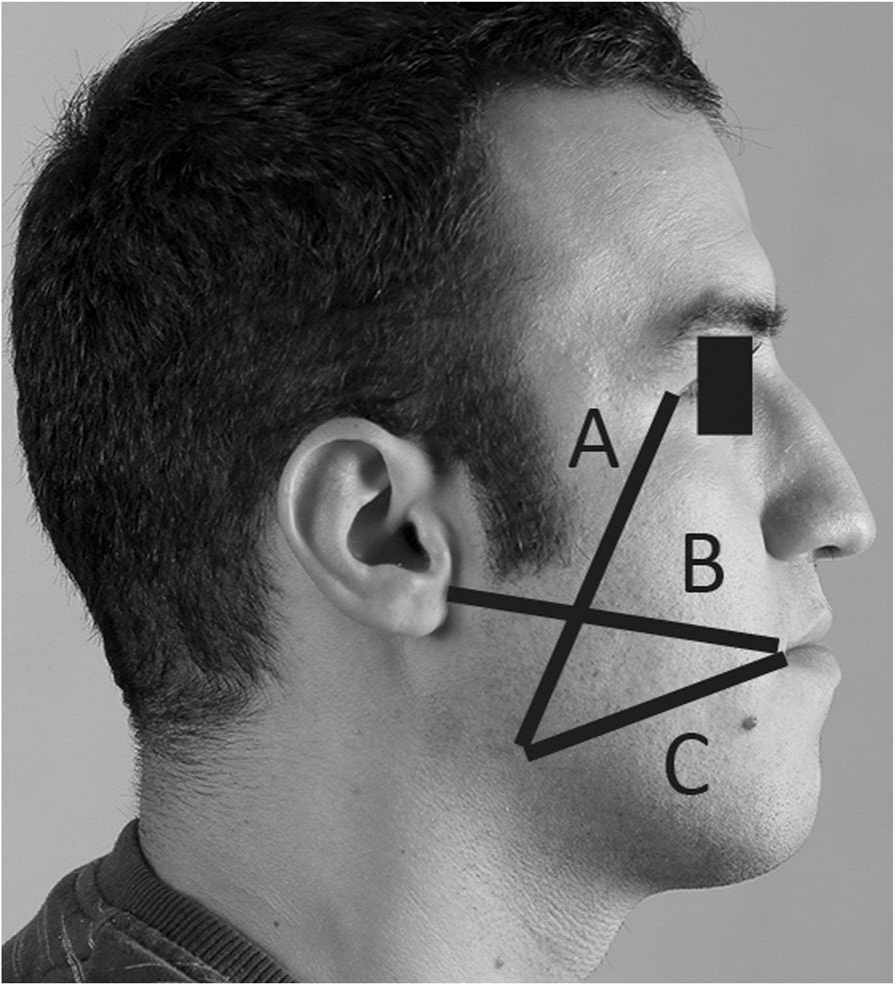
Postoperative edema linear demarcation measures. Line A is the vertical dimension to assess the swelling. Lines B and C are the assessment lines to evaluate the oblique and horizontal swelling, respectively

The patients had no pain at the surgical site (zero scores) before the surgery. The measurements were measured twice, and the average was established in the record sheet. These variables included the following:

1. The maximum mouth opening (MMO) measured by a digital caliper (Mitutoyo, Illinois, USA) as the distance from the central edge of the maxillary central incisor to the central edge of the central incisor of the mandible.
2. Facial width was estimated in three directions on a piece of dental floss and then measured by a ruler (Fig. 2). Horizontal distance: The distance between the corner of the mouth to the junction of the ear lobe on the same side and the floss followed the convexity of the cheek. Vertical distance: The distance between the outer canthus of the eye to the mandibular angle of the same side and a piece of floss followed the convexity of the face. Oblique distance: The distance from the corner of the mouth to the angle of the mandible on the same side and the piece of thread followed the possible convexity of the face (postoperative swelling). While performing these three measurements, the patient laid supine on the dental unit.
3. The depth of the gingival pocket in the distal of the second molar was measured by William’s probe (Juya Instruments PVT, Tehran, Iran). Three sizes of distobuccal, mid-distal, and distolingual were measured, and the average of these three numbers was recorded.
4. Attachment loss at the distal of the second molar was measured by William’s probe (Juya Instruments PVT, Tehran, Iran). Three sizes of distobuccal, mid-distal, and distolingual were measured, and the average of these three numbers was recorded.
5. The pain of the patient was recorded according to the visual analysis score (VAS).
6. Dry socket.

All the variables were measured and checked by three people (two dentists and one expert maxillofacial surgeon). The average value was used in the current study.

Patients were told to report the problem in the event of severe pain and discomfort. VAS forms were given to the patient to record the pain: 12 and 24 h after surgery and on days 2, 3, 4, 5, 6, and 7.

MMO and the amount of edema were measured and recorded 24 h after the surgery and on the 3rd and 7th mornings after the surgery. On the seventh day, the sutures were removed and the opened suture spontaneously without the patient’s intervention was examined.

Six months following the surgery, the amount of gingival attachment loss and pocket depth on the distal of the second molars were measured the same as before the surgery.

### Ethical consideration

All the patients filled out the consent forms. The procedures followed were following the ethical standards of the responsible committee of Semnan University of Medical Sciences with the ethical code IR.SEMUMS.REC.1397.157. This project was also registered to the Iranian Registry of Clinical Trials under the supervision of WHO with the ethical code IRCT201311263750N1.

### Statistical analysis

Data were analyzed using descriptive statistics (number and percentage) and diagrams in SPSS version 26 (IBM Corporation, Armonk, NY). The paired *t*-test and Wilcoxon tests were used to describe numerical variables. *P* value < 0.05 was considered as the significant level.

## Results

This randomized clinical trial was designed as a split-mouth double-blinded survey and executed in 2019 in Semnan, Iran. Twenty patients were included in the current study. The patients were between 20 and 29 years old (mean age of 24.17 ± 2.2). Twenty-one patients (52.5%) were female and 19 patients (47.5%) were male.

Regarding the mean pain of the patients in 12 h and the first day after the surgery, the average patients’ pain in the control side was significantly lower than that in the case side (Table 1).

**Table 1:** Pain perception following surgical extraction of the third molar

Follow-up period	Pain score in control side (mean ± SD)	Pain score in case side (mean ± SD)	*P* value
12 h post-surgery	8.1 ± 1.25	8.75 ± 1.46	0.041^a^
Day 1 post-surgery	6.7 ± 1.48	7.45 ± 1.21	0.02^a^
Day 2 post-surgery	4.95 ± 1.33	5.12 ± 1.53	0.59
Day 3 post-surgery	3.1 ± 1.61	3.25 ± 1.82	0.67
Day 4 post-surgery	1.32 ± 1.07	1.22 ± 1.12	0.76
Day 5 post-surgery	0.5 ± 0.84	0.48 ± 0.67	0.76
Day 6 post-surgery	0.3 ± 0.64	0.22 ± 0.53	0.49
Day 7 post-surgery	0.15 ± 0.48	0.05 ± 0.31	0.33

*SD* standard deviation

^a^Statistically significant

The mean vertical swelling in the patients of the two sides after the surgery was compared with each other. The results showed that on the first and third days after surgery, the rate of vertical swelling in the case side was significantly lower than in the control side. Also, the mean horizontal swelling of patients on two sides after the surgery was compared with each other, and on the first and third days after surgery, the horizontal swelling in the case side was significantly lower than in the control side. The mean of swelling in the oblique direction on the first and third days after the surgery was significantly lower in the case side (Table 2).

**Table 2:** Effects of the two different suturing techniques on facial edema following surgical extraction of the third molar

Variable	Time	Control side (mean ± SD), mm	Case side (mean ± SD), mm	*P* value
Vertical facial dimension (difference before the surgery)	Day 1	1.24 ± 2.63	0.8 ± 1.77	0.001^a^
	Day 3	6.9 ± 3.65	4.9 ± 3.42	0.001^a^
	Day 7	0.36 ± 2.17	0.2 ± 1.47	0.057
Horizontal facial dimension (difference before the surgery)	Day 1	3.2 ± 2.79	1.52 ± 1.86	0.001^a^
	Day 3	7.42 ± 3.94	5.05 ± 3.41	0.001^a^
	Day 7	0.12 ± 1.1	0.08 ± 0.93	0.07
Oblique facial dimension (difference before the surgery)	Day 1	2.97 ± 3.1	0.8 ± 1.4	0.001^a^
	Day 3	8.45 ± 3.37	5.07 ± 2.91	0.001^a^
	Day 7	1.1 ± 1.75	0.75 ± 1.6	0.411

*SD* standard deviation

^a^Statistically significant

The MMO rate in the control side was significantly more than in the case side (Table 3). The mean of pocket depth in the control side was significantly higher than in the case side. The mean rate of gingival attachment loss in the control side was significantly higher than that in the case side (Table 4).

**Table 3:** Maximum mouth opening (MMO) difference before and after the surgical process

Follow-up period	MMO in the control side (mean ± SD), mm	MMO in the case side, (mean ± SD), mm	*P* value
Day 1	18.03 ± 3.4	20.4 ± 2.69	0.001^a^
Day 3	15.38 ± 2.52	16.3 ± 2.65	0.001^a^
Day 7	1.38 ± 2.88	1.4 ± 2.49	0.97

*SD* standard deviation

^a^Statistically significant

**Table 4:** Periodontal status of the two sides 6 months after the surgery

Variable	Control side	Case side	*P* value
Pocket depth (mean ± SD) mm	3.26 ± 0.38	2.92 ± 0.35	0.001^a^
Attachment loss (mean ± SD), mm	0.94 ± 0.22	0.08 ± 0.23	0.01^a^

*SD* standard deviation

^a^Statistically significant

The frequency of postoperative dry socket was compared between the two sides, and the findings were not statistically significant (four cases in the control side versus one case in the case side). The incidence of wound dehiscence after the surgery in the control side was significantly more than in the case side (32.5% versus 12.5%; *P* value = 0.031).

## Discussion

Third molar surgery is the most common surgery in the field of oral and dental surgeries, and many patients are diagnosed and operated on by dentists to extract this tooth. Extensive research with the aim of improving a variety of complications, such as pain, swelling, trismus, bleeding, sensation, dry socket, and periodontal health of the second molar, is still being addressed, which shows the importance of this goal. In this study, it was attempted to compare a new suturing technique with simple interrupted sutures.

The findings of the current study showed that the pain was significantly less in 12 h (the morning after the surgery) and in the first 24 h after the surgery in the side routine suturing technique was used. Swelling and trismus were also significantly better in the single suture side on the first and third days after the surgery. In contrast, the pocket depth of the second molar and attachment loss were less in 6 months after the surgery in the side newly invented suture was used.

The most common method of wound closure in oral surgery is suturing. Various sutures are introduced and used in oral surgeries. The single interrupted suture is the most common suture used by surgeons after the extraction of the wisdom tooth. The degree of stretch of the wound edges after suturing is one of the important factors in the occurrence of inflammatory complications and of course the sensation after the surgery. In the new method presented in the current study, which is called tension breaking knot, a special concern is about how to close the wound without loosening the sutures. Although the innovative suture introduced in this study holds the edges of the wound tightly together and prevents their movement, and practically needs initial repair, the type of knot is such that it removes the tension from the edges of the wound and transfers it to the knots. As a result, the innovative suture, by keeping the clot in the tooth socket and preventing food impaction to the extraction site, reduces the pain following the surgery. Furthermore, an initial restoration and possibly a faster healing process are also effective in reducing inflammatory complications. Considering that there are contradictory results in studies related to secondary and primary repair in reducing complications, such as pain and swelling, and considering the advantages we mentioned for the innovative suture, in this study, we compare the innovative suture with the individual discontinuous suture.

Another important complication is periodontal defects at the distal of the second molar adjacent to the surgical site, which appear after the surgery for a long time. Unlike the postoperative inflammatory complications, such as pain, bleeding, and swelling, which are the main causes of discomfort and complaints of the patients after the surgery, the formation of a pocket and loss of distal gingival attachment of the second molar following the surgery are hidden to many patients or are not considered as an important issue. However, the presence of the pocket and the gingival attachment loss around the tooth prevents the patient from observing proper hygiene, and further increasing the depth of the pocket results in the same inability to maintain hygiene in a vicious cycle. Therefore, it is especially important to prevent these important complications [14, 15]. Cetinkaya et al. compared two common anchor sutures in the mandibular impact wisdom tooth surgery on the variables of gingival loss and the depth of the distal pocket of the second molar, with superior results of anchor suture after 6 months [2]. The anchor suture is faced with an increased inflammatory process due to the wicking effect of the suture around the second molar. Despite the anchor suture, the newly presented suturing technique helps in placing the gingival papilla in the right place. Alqahtani et al. [8] examined two types of flaps called envelope flaps and modified triangular flaps in terms of pain, swelling, and the depth of the distal pocket of the second molar following mandibular impact wisdom tooth surgery. The pocket depth was significantly less in the modified triangular flap. In contrast, the swelling in this type of flap was significantly more than the envelope flap. Although the pain was more in the modified triangular flap group, the results were not significant [8]. Modified flaps such as the technique used in Alqahtani et al.’s study may be harmful to the gingival margin and proceed the gingival recession around the second molar.

Considering the features mentioned about the innovative suture, it is hypothesized that this suture is superior to the usual suture in the amount of pocket depth and the attachment loss of the second molar. The important point of this study was to evaluate new suturing technique effects on the inflammatory variables and periodontal health of the second molar which are investigated in a few studies. Selecting the age range between 20 and 30 years old is because impacted third molar usually does not erupt after the age of 20 and the increased bone density after the age of 30 leads to surgery difficulties and periodontal problems after the surgery.

Also, although the innovative suture was predicted to excel in inflammatory factors, such as pain, swelling, and trismus due to the removal of traction from the wound edges and the prevention of food impaction, the failure to drain the inflammatory exudate from the surgical site was a more important factor, which made the variables in the control side better. Although pocket depth and attachment loss after 6 months were reduced significantly when the newly presented suture was used, these results were not significant. Besides, no dry socket was reported in the innovative suture side. The attachment loss difference was statistically significant, although this numerical difference may not be clinically significant. These limited differences between the two study groups may be due to the included patients’ mean age who were young and the bone regeneration progress and wound healing are way much better than older patients. The limitation of the current study was selecting younger patients since it is not allowed to extract the impacted third molar over 35 years old unless there is a significant indication. However, the pattern design of the new suturing technique may reduce the gingival recession also in the elderly which is needed to be evaluated in future studies. The clinical implications of the current suturing technique are the easy pattern and effective application in wound closure which make this helpful in implant surgery to have a water-tight wound closure.

## Conclusion

In conclusion, the newly presented suturing technique may be useful in reducing the periodontal complication of the distal of the second molar following the surgical procedure of the mandibular impacted third molar including the pocket formation and the attachment loss. By designing the special knot during the suturing the wound edges would be kept close together and prevent the gingival recession.

## Data Availability

Sharing is not applicable to this article since no dataset was generated or analyzed during the current study.

## Abbreviations

MMO: Maximum mouth opening
VAS: Visual analysis score
